# Supplementary material for: An open-source framework for large-scale, flexible evaluation of biomedical text mining systems
Source: J Biomed Discov Collab. 2008 Jan 29;3:1. doi: 10.1186/1747-5333-3-1 (PMC2276192; doi:10.1186/1747-5333-3-1)
Supplement: Additional file 1 — Evaluation platform source code. The additional file contains the software evaluation framework discussed in this paper. The most current release can be downloaded from SourceForge.net [1]. See the accompanying README file for instructions on installation and use. Also included in the distribution is a collection of UIMA wrappers for some commonly used BioNLP tools and annotated corpora. [file 1747-5333-3-1-S1.zip › bionlp_uima_repository-1.0.1/docs/api/edu/uchsc/ccp/uima/util/UIMA_Util.html]

UIMA\_Util (Center for Computational Pharmacology Util/UIMA API)


|  |  |  |
| --- | --- | --- |
| **Overview** \| **Package** \| **Class** \| **Tree** \| **Deprecated** \| **Index** \| **Help** | |  |
| **PREV CLASS**   **NEXT CLASS** | **FRAMES**    **NO FRAMES**     **All Classes** |  |
| SUMMARY: NESTED \| FIELD \| CONSTR \| METHOD | DETAIL: FIELD \| CONSTR \| METHOD |  |


---


## edu.uchsc.ccp.uima.util Class UIMA\_Util

```
java.lang.Object
  edu.uchsc.ccp.uima.util.UIMA_Util
```

---

``` public class UIMA_Util extends Object ```

This is a utility class for converting between UIMA annotations and TextAnnotations.

**Author:**
:   William A. Baumgartner, Jr.

---

**Constructor Summary**

`UIMA_Util()`


| **Method Summary** | |
| --- | --- |
| `static void` | `addCCPTextAnnotationToCCPClassMention(CCPTextAnnotation ccpTA, CCPClassMention ccpCM)` |
| `static void` | `addSlotValue(CCPClassMention ccpClassMention, String slotMentionName, String slotValue)` |
| `static Iterator<CCPTextAnnotation>` | `getAnnotationsEncompassingSpan(Span span, org.apache.uima.jcas.JCas jcas)` |
| `static Iterator<CCPTextAnnotation>` | `getAnnotationsEncompassingSpan(Span span, org.apache.uima.jcas.JCas jcas, int annotType)`             return all annotations that contain the input span |
| `static Iterator<CCPTextAnnotation>` | `getAnnotationsInBetweenSpans(Span upstreamSpan, Span downstreamSpan, org.apache.uima.jcas.JCas jcas)`             return annotations in between spans |
| `static Iterator<CCPTextAnnotation>` | `getAnnotationsWithinSpan(Span span, org.apache.uima.jcas.JCas jcas)` |
| `static Iterator` | `getAnnotationsWithinSpan(Span span, org.apache.uima.jcas.JCas jcas, int annotationType)`             return all annotations within the exact same span as the input span |
| `static Iterator<CCPTextAnnotation>` | `getAnnotationsWithSameStart(int startIndex, org.apache.uima.jcas.JCas jcas)`             return all annotations that start with the input startIndex |
| `static String` | `getDocumentID(org.apache.uima.jcas.JCas jcas)` |
| `static String` | `getFirstSlotValue(CCPNonComplexSlotMention ccpNonComplexSlotMention)` |
| `static List<CCPSlotMention>` | `getMultipleSlotMentionsByName(CCPClassMention ccpClassMention, String slotMentionName)` |
| `static List<CCPSlotMention>` | `getMultipleSlotMentionsByName(CCPTextAnnotation ccpTextAnnotation, String slotMentionName)` |
| `static Iterator<CCPTextAnnotation>` | `getOverlappingAnnotations(CCPTextAnnotation ccpTA, org.apache.uima.jcas.JCas jcas, int annotType)` |
| `static Iterator<CCPTextAnnotation>` | `getPrecedingAnnotations(int startIndex, int ccpAnnotationType, org.apache.uima.jcas.JCas jcas)` |
| `String` | `getSingleLineRepresentationOfCCPClassMention(CCPClassMention ccpCM)` |
| `String` | `getSingleLineRepresentationOfCCPComplexSlotMention(CCPComplexSlotMention ccpCSM)` |
| `String` | `getSingleLineRepresentationOfCCPNonComplexSlotMention(CCPNonComplexSlotMention ccpNCSM)` |
| `String` | `getSingleLineRepresentationOfCCPTextAnnotation(CCPTextAnnotation ccpTA, boolean deep)` |
| `static CCPSlotMention` | `getSlotMentionByName(CCPClassMention ccpClassMention, String slotMentionName)` |
| `static CCPSlotMention` | `getSlotMentionByName(CCPTextAnnotation ccpTextAnnotation, String slotMentionName)` |
| `List<TextAnnotation>` | `getTextAnnotationsFromJCas(org.apache.uima.jcas.JCas jcas)` |
| `static void` | `printCCPAnnotationSet(CCPAnnotationSet ccpAnnotationSet, PrintStream ps)` |
| `static void` | `printCCPAnnotator(CCPAnnotator ccpAnnotator, PrintStream ps)` |
| `static void` | `printCCPClassMention(CCPClassMention ccpClassMention, PrintStream ps, int indentLevel)` |
| `static void` | `printCCPComplexSlotMention(CCPComplexSlotMention ccpComplexSlotMention, PrintStream ps, int indentLevel)` |
| `static void` | `printCCPNonComplexSlotMention(CCPNonComplexSlotMention ccpNonComplexSlotMention, PrintStream ps, int indentLevel)` |
| `static void` | `printCCPTextAnnotation(CCPTextAnnotation ccpTextAnnotation, PrintStream ps)` |
| `void` | `putTextAnnotationsIntoJCas(org.apache.uima.jcas.JCas jcas, List<TextAnnotation> textAnnotations)` |
| `static void` | `setCCPPClassMentionForCCPTextAnnotation(CCPTextAnnotation ccpTA, CCPClassMention ccpCM)`             sets the class mention for the CCPTextAnnotation, and also links the CCPTextAnnotation with the CCPClassMention |
| `static void` | `setCCPTextAnnotationSpan(CCPTextAnnotation ccpTA, int spanStart, int spanEnd)`             this method resets the span for an annotation.. |
| `static void` | `swapAnnotationInfo(CCPTextAnnotation fromUIMA, CCPTextAnnotation toUIMA)`             Swap from UIMA Annotation to another UIMA Annotation |
| `static void` | `swapAnnotationInfo(CCPTextAnnotation fromUIMA, TextAnnotation toTA, org.apache.uima.jcas.JCas jcas)`             This method transfers general annotation info, i.e. |
| `static void` | `swapAnnotationInfo(TextAnnotation fromTA, CCPTextAnnotation toUIMA, org.apache.uima.jcas.JCas jcas)`             This method transfers general annotation info, i.e. |
| `static void` | `swapAnnotationSetInfo(AnnotationSet annotationSet, CCPAnnotationSet ccpAnnotationSet)` |
| `static void` | `swapAnnotationSetInfo(CCPAnnotationSet ccpAnnotationSet, AnnotationSet annotationSet)` |
| `static void` | `swapAnnotatorInfo(Annotator annotator, CCPAnnotator ccpAnnotator)` |
| `static void` | `swapAnnotatorInfo(CCPAnnotator ccpAnnotator, Annotator annotator)` |
| `static void` | `swapClassMentionInfo(CCPClassMention fromCM, CCPClassMention toCM)` |
| `static void` | `swapComplexSlotMentionInfo(CCPComplexSlotMention fromSM, CCPComplexSlotMention toSM)` |
| `static void` | `swapDocumentInfo(org.apache.uima.jcas.JCas fromJcas, GenericDocument toGD)` |
| `static void` | `swapNonComplexSlotMentionInfo(CCPNonComplexSlotMention fromSM, CCPNonComplexSlotMention toSM)` |
| `static boolean` | `validateCCPClassMention(CCPClassMention ccpCM)`             This method checks each slot mention associated with the input class mention for valid slot fillers. |
| `static boolean` | `validateCCPComplexSlotMention(CCPComplexSlotMention ccpCSM)`             This method checks for valid slot fillers of the input CCPComplexSlotMention, i.e. |
| `static boolean` | `validateCCPNonComplexSlotMention(CCPNonComplexSlotMention ccpNCSM)`             This method checks for valid slot fillers of the input CCPNonComplexSlotMention, i.e. |
| `static boolean` | `validateCCPSlotMention(CCPSlotMention ccpSM)`             This method determines the type of slot mention inputted, and calls either validateCCPComplexSlotMention() or validateCCPNonComplexSlotMention() |
| `static boolean` | `validateCCPTextAnnotation(CCPTextAnnotation ccpTA)`             This method provides a sanity check when constructing CCPTextAnnotations, and in particular the class mention structure from scratch. |

**Methods inherited from class java.lang.Object**

`clone, equals, finalize, getClass, hashCode, notify, notifyAll, toString, wait, wait, wait`

**Constructor Detail**

### UIMA\_Util

```
public UIMA_Util()
```


**Method Detail**

### getDocumentID

```
public static String getDocumentID(org.apache.uima.jcas.JCas jcas)
```

---


### swapDocumentInfo

```
public static void swapDocumentInfo(org.apache.uima.jcas.JCas fromJcas,
                                    GenericDocument toGD)
```

---


### swapAnnotationInfo

```
public static void swapAnnotationInfo(CCPTextAnnotation fromUIMA,
                                      TextAnnotation toTA,
                                      org.apache.uima.jcas.JCas jcas)
```

:   This method transfers general annotation info, i.e. span, annotator, etc. from a CCPTextAnnotation to a TextAnnotation

    :   **Parameters:**: `ccpAnnotation` -: `ta` -

---


### swapAnnotationInfo

```
public static void swapAnnotationInfo(CCPTextAnnotation fromUIMA,
                                      CCPTextAnnotation toUIMA)
                               throws org.apache.uima.cas.CASException
```

:   Swap from UIMA Annotation to another UIMA Annotation

    :   **Parameters:**: `fromUIMA` -: `toUIMA` -: `jcas` - **Throws:**: `org.apache.uima.cas.CASException`

---


### swapClassMentionInfo

```
public static void swapClassMentionInfo(CCPClassMention fromCM,
                                        CCPClassMention toCM)
                                 throws org.apache.uima.cas.CASException
```

:   **Throws:**: `org.apache.uima.cas.CASException`

---


### swapNonComplexSlotMentionInfo

```
public static void swapNonComplexSlotMentionInfo(CCPNonComplexSlotMention fromSM,
                                                 CCPNonComplexSlotMention toSM)
                                          throws org.apache.uima.cas.CASException
```

:   **Throws:**: `org.apache.uima.cas.CASException`

---


### swapComplexSlotMentionInfo

```
public static void swapComplexSlotMentionInfo(CCPComplexSlotMention fromSM,
                                              CCPComplexSlotMention toSM)
                                       throws org.apache.uima.cas.CASException
```

:   **Throws:**: `org.apache.uima.cas.CASException`

---


### swapAnnotationInfo

```
public static void swapAnnotationInfo(TextAnnotation fromTA,
                                      CCPTextAnnotation toUIMA,
                                      org.apache.uima.jcas.JCas jcas)
```

:   This method transfers general annotation info, i.e. span, annotator, etc. from a TextAnnotation to a CCPTextAnnotation.

    :   **Parameters:**: `ta` -: `ccpAnnotation` -

---


### swapAnnotatorInfo

```
public static void swapAnnotatorInfo(CCPAnnotator ccpAnnotator,
                                     Annotator annotator)
```

---


### swapAnnotatorInfo

```
public static void swapAnnotatorInfo(Annotator annotator,
                                     CCPAnnotator ccpAnnotator)
```

---


### swapAnnotationSetInfo

```
public static void swapAnnotationSetInfo(CCPAnnotationSet ccpAnnotationSet,
                                         AnnotationSet annotationSet)
```

---


### swapAnnotationSetInfo

```
public static void swapAnnotationSetInfo(AnnotationSet annotationSet,
                                         CCPAnnotationSet ccpAnnotationSet)
```

---


### getTextAnnotationsFromJCas

```
public List<TextAnnotation> getTextAnnotationsFromJCas(org.apache.uima.jcas.JCas jcas)
```

---


### putTextAnnotationsIntoJCas

```
public void putTextAnnotationsIntoJCas(org.apache.uima.jcas.JCas jcas,
                                       List<TextAnnotation> textAnnotations)
```

---


### printCCPAnnotator

```
public static void printCCPAnnotator(CCPAnnotator ccpAnnotator,
                                     PrintStream ps)
```

---


### printCCPAnnotationSet

```
public static void printCCPAnnotationSet(CCPAnnotationSet ccpAnnotationSet,
                                         PrintStream ps)
```

---


### getSingleLineRepresentationOfCCPTextAnnotation

```
public String getSingleLineRepresentationOfCCPTextAnnotation(CCPTextAnnotation ccpTA,
                                                             boolean deep)
```

---


### getSingleLineRepresentationOfCCPNonComplexSlotMention

```
public String getSingleLineRepresentationOfCCPNonComplexSlotMention(CCPNonComplexSlotMention ccpNCSM)
```

---


### getSingleLineRepresentationOfCCPComplexSlotMention

```
public String getSingleLineRepresentationOfCCPComplexSlotMention(CCPComplexSlotMention ccpCSM)
```

---


### getSingleLineRepresentationOfCCPClassMention

```
public String getSingleLineRepresentationOfCCPClassMention(CCPClassMention ccpCM)
```

---


### printCCPTextAnnotation

```
public static void printCCPTextAnnotation(CCPTextAnnotation ccpTextAnnotation,
                                          PrintStream ps)
```

---


### printCCPClassMention

```
public static void printCCPClassMention(CCPClassMention ccpClassMention,
                                        PrintStream ps,
                                        int indentLevel)
```

---


### printCCPComplexSlotMention

```
public static void printCCPComplexSlotMention(CCPComplexSlotMention ccpComplexSlotMention,
                                              PrintStream ps,
                                              int indentLevel)
```

---


### printCCPNonComplexSlotMention

```
public static void printCCPNonComplexSlotMention(CCPNonComplexSlotMention ccpNonComplexSlotMention,
                                                 PrintStream ps,
                                                 int indentLevel)
```

---


### getSlotMentionByName

```
public static CCPSlotMention getSlotMentionByName(CCPTextAnnotation ccpTextAnnotation,
                                                  String slotMentionName)
```

---


### getSlotMentionByName

```
public static CCPSlotMention getSlotMentionByName(CCPClassMention ccpClassMention,
                                                  String slotMentionName)
```

---


### getMultipleSlotMentionsByName

```
public static List<CCPSlotMention> getMultipleSlotMentionsByName(CCPTextAnnotation ccpTextAnnotation,
                                                                 String slotMentionName)
```

---


### getMultipleSlotMentionsByName

```
public static List<CCPSlotMention> getMultipleSlotMentionsByName(CCPClassMention ccpClassMention,
                                                                 String slotMentionName)
```

---


### getFirstSlotValue

```
public static String getFirstSlotValue(CCPNonComplexSlotMention ccpNonComplexSlotMention)
```

---


### addSlotValue

```
public static void addSlotValue(CCPClassMention ccpClassMention,
                                String slotMentionName,
                                String slotValue)
                         throws org.apache.uima.cas.CASException
```

:   **Throws:**: `org.apache.uima.cas.CASException`

---


### getAnnotationsWithinSpan

```
public static Iterator<CCPTextAnnotation> getAnnotationsWithinSpan(Span span,
                                                                   org.apache.uima.jcas.JCas jcas)
```

---


### getAnnotationsWithinSpan

```
public static Iterator getAnnotationsWithinSpan(Span span,
                                                org.apache.uima.jcas.JCas jcas,
                                                int annotationType)
```

:   return all annotations within the exact same span as the input span

    :   **Parameters:**: `span` -: `jcas` - **Returns:**

---


### getOverlappingAnnotations

```
public static Iterator<CCPTextAnnotation> getOverlappingAnnotations(CCPTextAnnotation ccpTA,
                                                                    org.apache.uima.jcas.JCas jcas,
                                                                    int annotType)
```

---


### getAnnotationsEncompassingSpan

```
public static Iterator<CCPTextAnnotation> getAnnotationsEncompassingSpan(Span span,
                                                                         org.apache.uima.jcas.JCas jcas)
```

---


### getAnnotationsEncompassingSpan

```
public static Iterator<CCPTextAnnotation> getAnnotationsEncompassingSpan(Span span,
                                                                         org.apache.uima.jcas.JCas jcas,
                                                                         int annotType)
```

:   return all annotations that contain the input span

    :   **Parameters:**: `span` -: `jcas` - **Returns:**

---


### getAnnotationsInBetweenSpans

```
public static Iterator<CCPTextAnnotation> getAnnotationsInBetweenSpans(Span upstreamSpan,
                                                                       Span downstreamSpan,
                                                                       org.apache.uima.jcas.JCas jcas)
```

:   return annotations in between spans

    :   **Parameters:**: `span` -: `jcas` - **Returns:**

---


### getAnnotationsWithSameStart

```
public static Iterator<CCPTextAnnotation> getAnnotationsWithSameStart(int startIndex,
                                                                      org.apache.uima.jcas.JCas jcas)
```

:   return all annotations that start with the input startIndex

    :   **Parameters:**: `startIndex` -: `jcas` - **Returns:**

---


### getPrecedingAnnotations

```
public static Iterator<CCPTextAnnotation> getPrecedingAnnotations(int startIndex,
                                                                  int ccpAnnotationType,
                                                                  org.apache.uima.jcas.JCas jcas)
```

---


### setCCPTextAnnotationSpan

```
public static void setCCPTextAnnotationSpan(CCPTextAnnotation ccpTA,
                                            int spanStart,
                                            int spanEnd)
                                     throws org.apache.uima.cas.CASException
```

:   this method resets the span for an annotation.. this includes the Span FSArray

    :   **Parameters:**: `ccpTA` -: `spanStart` -: `spanEnd` - **Throws:**: `org.apache.uima.cas.CASException`

---


### setCCPPClassMentionForCCPTextAnnotation

```
public static void setCCPPClassMentionForCCPTextAnnotation(CCPTextAnnotation ccpTA,
                                                           CCPClassMention ccpCM)
                                                    throws org.apache.uima.cas.CASException
```

:   sets the class mention for the CCPTextAnnotation, and also links the CCPTextAnnotation with the CCPClassMention

    :   **Parameters:**: `ccpTA` -: `ccpCM` - **Throws:**: `org.apache.uima.cas.CASException`

---


### addCCPTextAnnotationToCCPClassMention

```
public static void addCCPTextAnnotationToCCPClassMention(CCPTextAnnotation ccpTA,
                                                         CCPClassMention ccpCM)
                                                  throws org.apache.uima.cas.CASException
```

:   **Throws:**: `org.apache.uima.cas.CASException`

---


### validateCCPTextAnnotation

```
public static boolean validateCCPTextAnnotation(CCPTextAnnotation ccpTA)
```

:   This method provides a sanity check when constructing CCPTextAnnotations, and in particular the class mention structure from scratch. Slots are
    checked for appropriate filler, and error messages are raised if an invalid slot filler is discovered. True is returned if the
    CCPTextAnnotation has a valid mention structure, false otherwise.

    :   **Parameters:**: `ccpTA` - **Returns:**

---


### validateCCPClassMention

```
public static boolean validateCCPClassMention(CCPClassMention ccpCM)
```

:   This method checks each slot mention associated with the input class mention for valid slot fillers. True is returned if the CCPClassMention
    has a valid mention structure, false otherwise.

    :   **Parameters:**: `ccpTA` - **Returns:**

---


### validateCCPSlotMention

```
public static boolean validateCCPSlotMention(CCPSlotMention ccpSM)
```

:   This method determines the type of slot mention inputted, and calls either validateCCPComplexSlotMention() or
    validateCCPNonComplexSlotMention()

    :   **Parameters:**: `ccpSM` - **Returns:**

---


### validateCCPComplexSlotMention

```
public static boolean validateCCPComplexSlotMention(CCPComplexSlotMention ccpCSM)
```

:   This method checks for valid slot fillers of the input CCPComplexSlotMention, i.e. slot fillers must be valid CCPClassMentions. True is
    returned if the CCPClassMention has a valid mention structure, false otherwise.

    :   **Parameters:**: `ccpTA` - **Returns:**

---


### validateCCPNonComplexSlotMention

```
public static boolean validateCCPNonComplexSlotMention(CCPNonComplexSlotMention ccpNCSM)
```

:   This method checks for valid slot fillers of the input CCPNonComplexSlotMention, i.e. slot fillers must be Strings. This method is not
    necessary in truth because the StringArray object of each CCPNonComplexSlotMention will force the slot values to be strings, but is included
    for completeness.

    :   **Parameters:**: `ccpTA` - **Returns:**


---


|  |  |  |
| --- | --- | --- |
| **Overview** \| **Package** \| **Class** \| **Tree** \| **Deprecated** \| **Index** \| **Help** | |  |
| **PREV CLASS**   **NEXT CLASS** | **FRAMES**    **NO FRAMES**     **All Classes** |  |
| SUMMARY: NESTED \| FIELD \| CONSTR \| METHOD | DETAIL: FIELD \| CONSTR \| METHOD |  |


---
